# Notes and News

**Published:** 1899-08-05

**Authors:** 


### NOTES AND NEWS.

(Collected and Communicated.)

It is interesting to learn that out of 7,553 inquests held last year in the county of London only nine were held in public-houses. This is a great improvement.

Mr. Wyndham's farewell performance at the Criterion Theatre, given in aid of the Prince of Wales's Hospital Fund for London, produced the substantial sum of £1,485.

The present outbreak of the plague at Poona has carried off more than one European victim. Miss Bartley, who was attacked during the last week in July, has succumbed, and four other Europeans are now suffering from the disease. The total number of deaths exceeds 260. At Hong Kong an improvement is reported.

The Royal Society for the Protecti on of Life from Fire have just presented illuminated addresses to Dr. William Riddell Matthews, senior medical officer, Mr. W. Morrice, chief attendant, and Mr. W. Walker, of Aberdeen, in recognition of the prompt and efficient aid rendered by them at considerable personal danger in rescuing the patients on the occasion of a fire at the Royal Asylum, Aberdeen, a few months ago.

The newest poacher on medical preserves is the "Medical Scientific Botanist." Under this title a clerical gentleman named Stephen Maguth set up practice at Wandsworth, and treated a boy suffering from diphtheria. The boy died, and Mr. Maguth was prosecuted for treating him without adequate knowledge and for giving him improper medicines. He was convicted of manslaughter, but, on a recommendation to mercy, was sentenced to two months' imprisonment, without hard labour.

As an instance of the modern tendency to take one-sided views it may be mentioned that a New York scientist, Dr. Romane Curtis, formerly professor of bacteriology at the College of Surgeons and Physicians at Chicago, has announced his conviction that under the best hygienic conditions human beings should be able to live for a thousand years. The Professor regards the study of bacteriology for the purpose of destroying harmful bacteria as the means of securing longevity for the race. Whether an offer of a life of 1,000 years would be acceptable to many people is quite an open question.

The first expedition for the investigation of malaria in connection with the Liverpool School of Tropical Diseases sailed last Saturday for Sierra Leone. Major Ross is in charge of the party, which will afterwards visit Accra.

An outbreak of yellow fever, originating at the Soldiers' Home at Hampton, Virginia, in the United States, is causing great alarm. The whole district has been placed in quarantine, and the Government is taking vigorous measures to prevent the spread of the disease. Over 30 cases have been reported, and several deaths. It is believed that infection was brought by a soldier who had visited Santiago, and returned suffering from fever, which was diagnosed as malarial.

Sir Dyce Duckworth, presiding at the annual meeting of the Burial Reform Association, urged the desirability of simple, economical, and sanitary modes of burial. In regard to the lavish use of flowers at present-day funerals, he said that the fact that people occasionally put out appeals to their friends for no flowers indicated that there was a feeling in favour of a return to more simple and inexpensive ways. He was strongly in favour of the system of earth to earth burial.

The hon. secretaries of the Prince of Wales's Hospital Fund for London write that they have received complaints from annual subscribers to this Fund that they do not know to whom to send their subscriptions. As it is impossible for them to send reminders to those who have given indirectly, they ask us to make known through our paper that all subscriptions should be sent to the Hon. Secretaries, Prince of Wales's Hospital Fund, Bank of England, London, E.C., by whom they will be acknowledged.

In answer to a question in the House of Commons, Mr. Wyndham recently stated that the provision of chiropodists, for the purpose of attending to our soldiers' feet, had been under the careful consideration of the military authorities. As an experiment some men of the Royal Army Medical Corps were last year instructed in chiropody, and a class for a certain number of non-commissioned officers was subsequently formed at Aldershot. The experiment was successful, and its extension was, he said, under consideration. We would suggest that the expense which is incurred should be borne by the bootmaking branch of the army clothing department.

We understand that with the object of meeting the difficulty which at present exists in the early detection and treatment of mental diseases and kindred nervous affections, arrangements have been made at the Northamptonshire Asylum to afford a free consultation with the doctors of the asylum to those whose symptoms suggest incipient mental disease. The persons so applying will be regarded as out-patients of the asylum, and no expense will be incurred by them except the cost price of such medicines as the medical officers may think fit to supply. So, little by little, are we wearing down, on the medical side, the hard and fast distinctions between sanity and lunacy which have been so long maintained, and which the provisions of the law as to certificates have tended to crystallise upon lunacy practice.

